# The Multiple Roles of Glucose-6-Phosphate Dehydrogenase in Tumorigenesis and Cancer Chemoresistance

**DOI:** 10.3390/life12020271

**Published:** 2022-02-12

**Authors:** Jiaqi Song, Huanran Sun, Shuai Zhang, Changliang Shan

**Affiliations:** 1 State Key Laboratory of Medicinal Chemical Biology, College of Pharmacy and Tianjin Key Laboratory of Molecular Drug Research, Nankai University, Tianjin 300350, China; 2120201185@mail.nankai.edu.cn (J.S.); 2120201192@mail.nankai.edu.cn (H.S.); 2 School of Integrative Medicine, Tianjin University of Traditional Chinese Medicine, Tianjin 301617, China; 3 State Key Laboratory of Drug Research, Shanghai Institute of Materia Medica, Chinese Academy of Sciences, Shanghai 201203, China

**Keywords:** G6PD, tumorigenesis, chemotherapy resistance, inhibitor, metabolism, enzyme

## Abstract

The pentose phosphate pathway (PPP) is a branch from glycolysis that begins from glucose-6-phosphate (G6P) and ends up with fructose-6-phosphate (F6P) and glyceraldehyde-3-phosphate (GADP). Its primary physiological significance is to provide nicotinamide adenine dinucleotide phosphate (NADPH) and nucleotides for vital activities such as reactive oxygen species (ROS) defense and DNA synthesis. Glucose-6-phosphate dehydrogenase (G6PD) is a housekeeping protein with 514 amino acids that is also the rate-limiting enzyme of PPP, catalyzing G6P into 6-phosphogluconolactone (6PGL) and producing the first NADPH of this pathway. Increasing evidence indicates that G6PD is upregulated in diverse cancers, and this dysfunction influences DNA synthesis, DNA repair, cell cycle regulation and redox homeostasis, which provides advantageous conditions for cancer cell growth, epithelial-mesenchymal transition (EMT), invasion, metastasis and chemoresistance. Thus, targeting G6PD by inhibitors has been shown as a promising strategy in treating cancer and reversing chemotherapeutic resistance. In this review, we will summarize the existing knowledge concerning G6PD and discuss its role, regulation and inhibitors in cancer development and chemotherapy resistance.

## 1. Introduction

Basic metabolism, including lipid metabolism, glucose metabolism, protein metabolism and so on, is a basic characteristic of life activity that is essential in achieving material interchange, self-renewal and homeostasis maintenance. Glucose metabolism is one of the major energy sources whose complete oxidation, including glycolysis and the tricarboxylic acid cycle (TCA cycle), can release 2840 kJ of energy. Glucose absorbed extracellularly or transformed from other macromolecules is one of the primary carbon sources, and its catabolism mainly includes glycolysis, the TCA cycle and the pentose phosphate pathway (PPP). The catabolism of glucose not only provides nicotinamide adenine dinucleotide phosphate (NADPH), nucleotides and ATP but also supports lipid and amino acids synthesis for a variety of biological activities. PPP, also known as pentose phosphate shunt, is unlike glycolysis and the TCA cycle, because ATP is not produced in this pathway. Instead, it is the main source of NADPH, which is the primary reducing equivalent source for reactive oxygen species (ROS) defense and DNA synthesis. Glucose-6-phosphate dehydrogenase (G6PD) is the first and the rate-limiting enzyme of PPP, which has many essential biochemical functions. G6PD catalyzes glucose-6-phosphate (G6P) into 6-phosphogluconolactone (6PGL) with NADPH production. The dysfunction of G6PD is involved in many diseases, among which cancer is the most common ones. In this review, we will discuss the characteristics of G6PD, roles of G6PD in the development of cancer, inhibitors of G6PD and the mechanisms behind chemotherapy resistance caused by G6PD.

## 2. Historical Perspective of G6PD

Differential glucose metabolism between normal and tumor cells was observed by Otto Warburg in the early 19th century, which uncovered the research on cancer metabolism [1] In the 1930s, Warburg noticed that, in addition to the glycolytic cascade, G6P could also be metabolized by another oxidative pathway in which NADP^+^ was used for the oxidation of G6P. It was not until the 1950s, however, that the entire PPP was elucidated by Frank Dickens, Bernard Horecker, Fritz Lipmann and Efraim Racker, in which G6PD was demonstrated as the first enzyme of this pathway [2]. In 1956, human G6PD was first discovered by Carson et al., and its function was to produce NADPH for reduction [3]. In the same year, a G6PD deficiency was discovered in hemolytic anemia erythrocytes because of its low activity [4]. Further, a cloning study demonstrated that mutations existed on G6PD. The research on G6PD deficiency and its role in cancer began in 1965 when Beaconsfield was studying the relationship between areas and G6PD deficiency and G6PD-related cancer [5]. The cDNA of human G6PD was first cloned by Persico et al. in 1986, and the entire amino acid sequence was also uncovered, making the first step for the structural analysis of G6PD variants and beginning to know the enzyme features [6]. The structure of the G6PD protein was first observed in 1994, showing that this protein is a dimer [7], and there was no coenzyme binding sites except for catalytic sites. However, this protein was separated from *Leuconostoc mesenteroides,* whose glycolysis pathway was not complete [8,9]. G6PD separated from *Leuconostoc mesenteroides* can use both NAD^+^ and NADP^+^ [10,11] as coenzyme, which is different from advanced living organisms [12,13]. It took a long time and much effort to obtain the human G6PD tetramer due to its microheterogeneity. Margaret J. Adams and her colleagues obtained the crystallization [14], and in the following year, the human G6PD tetramer was revealed from a recombinant protein of the Canton variant [14]. This discovery solved the important question of whether the second NADP^+^ existed.

## 3. Characteristics of G6PD

**Figure 1:**
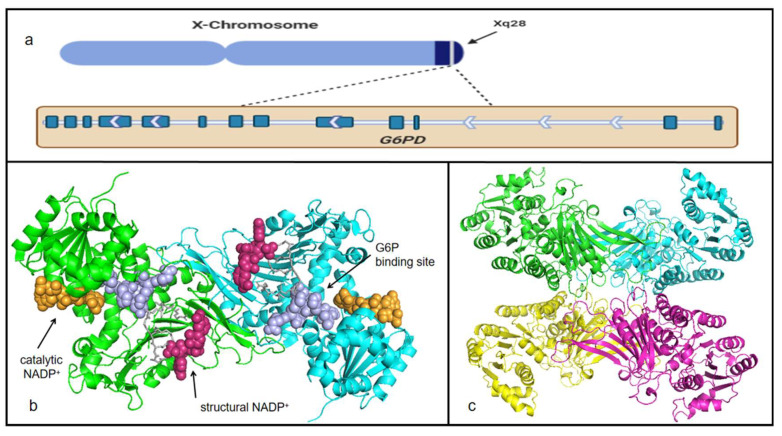
*G6PD* location on the chromosome and G6PD protein structure. (**a**) G6PD location is near the telomeric region of the distal arm. The wide, deep blue represents exons, and the arrows means the order of exons and introns. The first exon and intron are located on the right. For the protein structure, different colors present different peptides. Tertiary structures are downloaded from the website SWISS-MODEL and presented in the software PyMOL. (**b**) Dimer (a PDB:2bh9.1) of G6PD. Orange dots construct catalytic NADP^+^—binding sites; purple dots construct G6P-binding sites; pink dots construct structural NADP^+^—binding sites; gray sticks present sites Asn 363-Lys 366 and Arg 393-Gln 395. (**c**) Tetramer (b PDB:2bhl.1) of G6PD.

Given the fact that G6PD a typical housekeeping protein, it is expressed in all kinds of tissues and cells [15]. The gene encoding G6PD belongs to the X chromosome, and the location is near the telomeric region of the distal arm [16] (Figure 1a). The functions and structure of *G6PD* have been confirmed. The total length of this gene is about 18.5 kb, which includes 13 exons and 12 introns, and the last exon contains a terminator [16] (Figure 1a). The promoter of *G6PD* has been confirmed to be located at CpG land, and it has a sequence of TTAAAT, as well as a great number of stimulatory protein 1 (Sp1) elements, which is especially important for the activity of the promoter [16]. Exon and intron numbers and the exon sizes and sequences are conserved in higher eukaryotes [16]. The 5′-UTR of *G6PD* includes ATG codons that are out of the translation frame; additionally, the first ATG triplet upstream to a long open reading frame was judged to be the translation initiation site, and it was assigned position No. 1 [6]. mRNA produced by *G6PD* is 1545 bp [6]. The *G6PD* gene product is a 59-KD protein with 514 amino acids [6].

Human G6PD is a kind of NADP^+^-dependent catalytic enzyme. Secondary and tertiary structures have been revealed in the past few decades. Persico et al. determined the primary structure of G6PD for the first time using the sequence of full-length cDNA clones [6]. Ten years later, the structure and crystal of the mutant ΔG6PD enzyme, which has similar kinetic properties to those of the wild-type enzyme but is more thermostable, was demonstrated by Au et al. [14] The general tertiary structure of G6PD is a tetramer complex, which is constructed predominantly by a hydrophilic dimer–dimer complex [17] (Figure 1c). Each dimer contains two substrate sites and four NADP^+^—binding sites, two of which act as structural NADP^+^—binding sites, and the others act as catalytic NADP^+^—binding sites [6] (Figure 1b). According to the statistics provided by Wang et al., structural NADP^+^ tightly bound to the corresponding sites, not being removed by the usual dialysis and chromatographic procedures, and the true role of this NADP^+^ is believed to maintain long-term stability [18]. Catalytic NADP^+^—binding sites are those that loosely bound NADP^+^ or NADPH and were easily removed by dialysis [19]. Though tightly bound exists, structural NADP^+^ still can be removed in the form of NADPH when G6P binds to the substrate site. Once NADPH formed, it is easy to drop out of the structural NADP^+^—binding sites. Mechanistically, the presence of G6P will change the location of one or two amino acid residues (Asn 363-Lys 366 and Arg 393-Gln 395), each of which spans from the substrate site to the structural NADP^+^—binding site to the other [17,18,20]. Numerous diseases caused by a deficiency of G6PD affect people worldwide, and a high proportion of the clinical mutations are related to this structural NADP^+^ site [18,21,22,23]. Monomer is inactive form; however, dimer and tetramer are catalytically functional forms of G6PD [14]. The human enzyme is in a dimer↔tetramer equilibrium, and its stability is dependent on the NADP^+^ concentration [14]. Conversion towards the dimer happens under high pH conditions [24]. In addition, EDTA, NADPH and G6P favor disruption of the dimer, whereas NADP^+^ or certain metal ions favor the tetramer [14]. Additionally, the substrate-binding site and the dinucleotide-binding fingerprint are conserved in human G6PD with the sequences RIDHYLGKE (residues 198–206) and GxxGGDLA (residues 38–44), respectively [17].

## 4. G6PD and Cancer

Cancer is one of the most common diseases in the world, and it remains a leading cause of death worldwide every year. The causes of cancer are complicated, but common features still exist between different kinds of cancer. One of the hallmarks of cancer is reprogrammed energy metabolism [25], and decades ago, Warburg noticed that cancer cells were prone to metabolizing glucose through glycolysis with increased lactate production rather than the oxidative phosphorylation pathway, even in the presence of oxygen, known as the Warburg effect [26]. PPP flux is a main metabolic pathway of glucose, and the dysregulation of proteins in this pathway may be involved in the development of cancer. G6PD as the first and rate-limiting enzyme of PPP also plays an important role in the development of cancer. Indeed, G6PD is upregulated in many cancers. Besides, G6PD overexpression is associated with the degree and stage of cancer, including the size of the tumor, depth of invasion, lymph node metastasis, distant metastasis, TNM stage, and survival rate [27]. Rapid growth and proliferation demand massive biosynthesis and protection from the severe environment. Cancer cells hijack the intracellular metabolic pathway to maintain rapid replication and promote the synthesis of enormous essential cellular components. PPP plays a vital role in metabolism, which can provide both R-5-P for the synthesis nucleotide and NADPH for the redox equilibrium. The overexpression of G6PD influences DNA synthesis, DNA repair, cell cycle regulation, redox equilibrium, proliferation, EMT, invasion and metastasis to provide an advantageous condition for cancer cells [28,29,30,31,32]. What is more, G6PD also acts as a biomarker in diverse cancer in reflecting the clinical prognosis and chemoresistance.

## 5. The Regulation of G6PD in Cancer

The expression and activity of G6PD are tightly regulated, through transcription, post-transcription, translation, post-translation modification and interactions with other proteins [33] (Table 1).

### 5.1. Impacts on Cancer at Transcriptional Level

**Figure 2:**
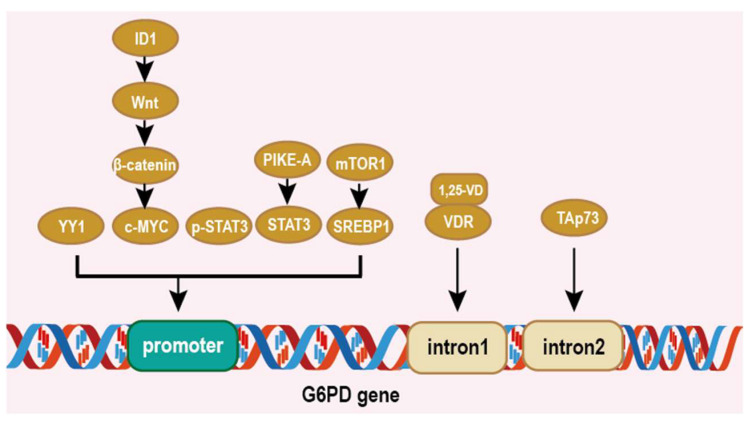
Regulation of G6PD at the transcriptional level. Elliptical shape represents the protein. Square shape represents the gene elements on *G6PD*. YY1, c-MYC, p-STAT3, STAT3, SREBP, 1,25VD/VDR and TAp73 bind to different parts of the G6PD gene to regulate G6PD expression.

Networks regulating G6PD are complicated in cancer cells and there are multiple cis/trans-regulatory elements controlling *G6PD* expression [43,44,45]. Simmons et al. reported that the 1, 25-VD/vitamin D receptor (VDR) binds to the vitamin D response elements (VDREs) located at the first intron of the G6PD genome to enhance the G6PD mRNA level [43]. Wu et al. suggested that the transcription factor yin yang 1 (YY1) also positively regulates G6PD at the transcriptional level by directly binding to the G6PD promoter, thus activating PPP and causing metabolic reprogramming [44]. Yin et al. found that c-MYC regulated by the ID1/Wnt/β-catenin axis binds to the promoter of *G6PD* and promotes G6PD transcription and encourages tumor proliferation and oxaliplatin chemoresistance in hepatocellular carcinoma (HCC) [45]. The Signal Transducer and Activator of Transcription 3 (STAT3) is a transcription factor that is upregulated in many cancers. Zhang et al. found that p-STAT3 activated G6PD gene expression via binding to the G6PD promoter [46]. What is more, Sun et al.’s study showed that PIKE-A induces the expression of G6PD through the binding of STAT3 at the *G6PD* promoter, thereby promoted glioblastoma cell growth [47]. There have been studies that revealed that the SREBP1 element exists on the *G6PD* promoter and increases its expression, which is regulated by mTOR1 [48,49]. TAp73, a structural homolog of the tumor suppressor p53, is frequently upregulated in human tumors [31,50]. TAp73 enhances the expression of G6PD by directly binding to the p53 family protein response element (RE) on the second intron of *G6PD* [31] (Figure 2). These studies indicate that a regulation expression of G6PD at the transcription level and targeting transcription factors maybe a promising therapeutic method.

### 5.2. Impacts on Cancer at Post-Transcriptional and Translational Level

**Figure 3:**
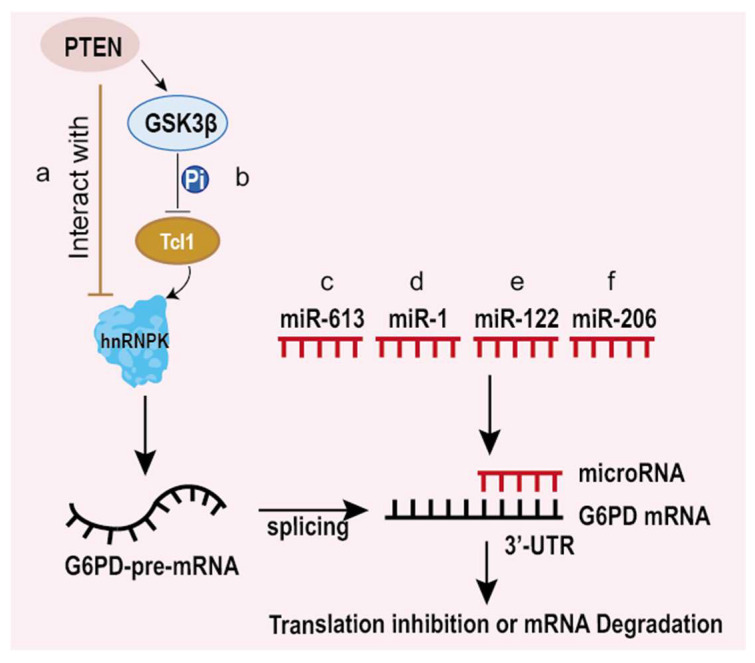
Regulation of G6PD at the post-transcriptional and translational levels. PTEN decreased G6PD expression by inhibiting its pre-mRNA splicing through not only interacting with hnRNPK (**a**)but inactivating Tcl1 through glycogen synthase kinase-3β (GSK3β)-mediated phosphorylation (**b**). MicroRNAs binds to G6PD mRNA to inhibit its translation or degradation. (**c**) miR-613 binds to the 3′-UTR of G6PD mRNA and promotes the degradation of G6PD mRNA. (**d**) miR-1 binds to the 3′-UTR of G6PD mRNA and promotes the degradation of G6PD mRNA or inhibition of G6PD translation. (**e**) miR-122 binds to the 3′-UTR of G6PD mRNA and promotes the degradation of G6PD mRNA. (**f**) miR-206 binds to the 3′-UTR of G6PD mRNA and promotes the degradation of G6PD mRNA.

Generally, eukaryotic genes are split genes containing introns and exons. Therefore, mRNA from the DNA template usually needs specific splicing for functional protein translation [51]. According to the work of Hong et al., T-cell leukemia 1 (Tcl1) can promote G6PD pre-mRNA splicing and protein expression by interacting with heterogeneous nuclear ribonucleoprotein (hnRNPK), finally leading to the development of liver cancer (Figure 3a). In addition, the phosphatase and tensin homolog (PTEN) decreased G6PD expression by inhibiting its pre-mRNA splicing through not only interacting with hnRNPK but inactivating Tcl1 through glycogen synthase kinase-3β (GSK3β)-mediated phosphorylation [52] (Figure 3b). This illustrates that PTEN controls G6PD expression through two steps by inhibiting G6PD pre-mRNA splicing, which suggests that G6PD is a key regulatory factor of PTEN in controlling tumor progression and implies that G6PD is a valuable target for anticancer treatments.

MicroRNAs (miRNAs) are small endogenous non-coding RNAs that regulate gene expressions by directly binding to mRNA to suppress the translation or degrade mRNA. Recently, there has been a growing body of evidence revealing that micro RNAs change metabolism, especially aerobic glycolysis to regulate tumorigenesis. A few microRNAs can bind to mRNA of G6PD to degrade *G6PD* mRNA, influencing the proliferation, migration and invasion of cancer cells. Some kinds of microRNAs play a vital role in regulating the translation of G6PD [28,42,53,54,55,56], which is also involved in controlling cancer progression.

In esophageal squamous cell carcinoma (ESCC), Su et al. found that miR-613 suppresses migration and invasion by decreasing the G6PD expression. Furthermore, they revealed that miR-613 directly binds to the 3′-UTR region of G6PD mRNA, leading to decreased G6PD mRNA [28] (Figure 3c). Hu et al. [53] and He et al. [42] found that miR-1 downregulates endogenous G6PD by directly binding to the 3′-UTR region and inhibiting proliferation and promoting apoptosis in high-risk papillomavirus-associated human cervical cancer and pituitary tumor cells (Figure 3d). Statistics from Barajas et al. [54] displayed for the first time that there are two conservative binding sites for combinations with miR-122 on the 3′-UTR region of G6PD mRNA in liver cancer cells, and interactions between miR-122 and G6PD mRNA reduce the G6PD mRNA and protein levels (Figure 3e). In HR-HPV-positive cervical cancer cells, miR-206 targets the 3′-UTR of G6PD mRNA and blocks its expression as well [55]. Besides, miR-206 is directly responsible for downregulating G6PD, which influences the growth of rhabdomyosarcoma cells [56] (Figure 3f). Thus, these results indicate that G6PD may serve as a therapeutic target for cancer treatment. In addition, micro RNAs not only downregulate G6PD expression individually but can also show a synergistic effect on inhibiting G6PD [54].

### 5.3. Impacts on Cancer at Post-Translational Modification Level

**Figure 4:**
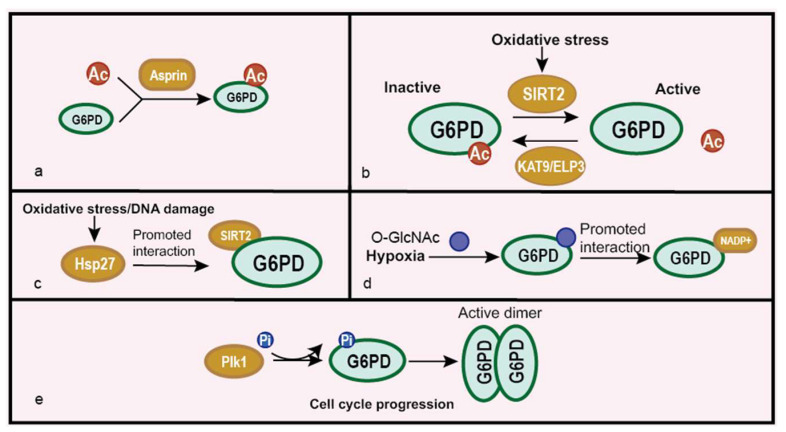
Regulation of G6PD at the post-translational modification level. (**a**) Aspirin acetylates G6PD and inhibits its activity. (**b**) Oxidative stress regulates G6PD acetylation by SIRT2 and KAT9/ELP3. (**c**) Hsp27 promotes the interaction between SIRT2 and G6PD, enhancing G6PD deacetylation caused by SIRT2 under oxidative stress or the DNA damage condition. (**d**) O-GlcNAcylation activates G6PD activity by promoting the interaction between G6PD and its co-factor NADPH under hypoxia. (**e**) Plk1 promotes the phosphorylation of G6PD to stimulate dimer formation during cell cycle progression.

In eukaryotes, post-translational modifications decide the activities and functions of proteins, which also exist in G6PD [57,58,59,60,61]. G6PD acetylation inhibits its function, while deacetylation reverses it [32,57,58,59]. Aspirin can acetylate G6PD at many functional sites and inactivates G6PD in HCT116 cells. The mass spectrometry analysis revealed that 14 lysine residues were acetylated which include essential catalysis sites K235 (in isoform *a*) and K205 (in isoform *b*). Acetylation of these two pivotal sites prevents the combination between G6PD and its substrate G-6-P, and acetylation at other lysine residues may decrease the activity of G6PD, leading to the decreased synthesis of ribose sugars and NADPH [57] (Figure 4a). According to the work of Xu et al., silent information regulator 2 (SIRT2) deacetylates G6PD at lysine 403 (K403) and consequently activates it to support the proliferation and clonogenic activity of leukemia cells. Chemical inhibitors against SIRT2 suppress G6PD activity, leading to the reduced cell proliferation of leukemia cells [58]. Elongator acetyltransferase complex subunit 3 (KAT9/ELP3) is identified as a potential acetyltransferase of G6PD, which participates in G6PD K403 acetylation [59] (Figure 4b). Besides, the work of Ye et al. suggests that the binding between G6PD and SIRT2 is enhanced by heat shock protein 27 (Hsp27), leading to the deacetylation and activation of G6PD [32] (Figure 4c). These studies indicate that the acetylation state of G6PD is important in metabolic reprogramming and tumor inhibition or proliferation. O-GlcNAcylation modification also actives G6PD, which can enhance the binding affinity of NADP^+^ to G6PD and form a higher oligomeric state [60] (Figure 4d). The enhanced activity of G6PD supports cell survival under oxidative stress, and this indicates that O-GlcNAcylation is a key metabolic regulator of the glucose flux. The phosphorylation of G6PD also plays a vital role in regulating G6PD activity. Polo-like kinase1 (Plk1) can bind to and phosphorylate G6PD to positively affect the formation of an active dimer promoting cell cycle progression [61] (Figure 4e). Other modification enzymes need to clarify its role on G6PD and their effects in cancer development. What is more, the additional modifications of G6PD and their function warrant further investigation. Whether modification crosstalk exists on G6PD is still an open question.

### 5.4. Impacts on Cancer under Interaction with Other Proteins

**Figure 5:**
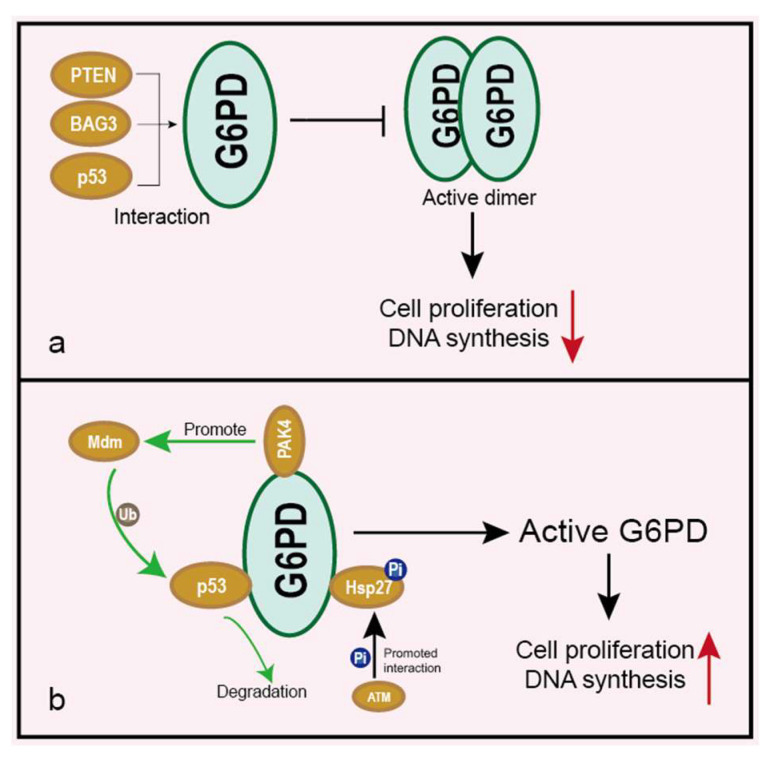
Interactions between G6PD and other proteins. Elliptical shape represents a protein. Circle shape represents post-translational modification. (**a**) Proteins such as PTEN, BAG3 or p53 bind to G6PD and inhibit G6PD active dimer formation, leading to the inhibition of cell proliferation and downregulation of DNA synthesis. (**b**) G6PD is activated through binding with Hsp27 and separating with p53, resulting in an enhanced cell proliferation rate and DNA synthesis. Hsp27 can be phosphorylated by ATM. p53 is ubiquitinated and degraded by the PAK4 pathway.

It has been reported that many proteins bind to G6PD, and regulatory interactions with G6PD mainly inhibit the formation of the active oligomeric state [40,62]. Bcl-2 associated athanogene 3 (BAG3) directly interacts with G6PD and consequently inhibits its dimerization and activity, leading to suppression of the PPP flux and the proliferation of hepatocellular carcinomas (HCCs) [40] (Figure 5a). p53 binds to G6PD and prevents the formation of the active dimer, thus inhibiting the PPP flux [62] (Figure 5a). p21-activated kinase 4 (PAK4) can also interact with G6PD and increase G6PD activity via enhancing Mdm2-mediated p53 ubiquitination degradation, resulting in increased colon cancer cell growth [63] (Figure 5b). As mentioned above, PTEN is closely associated with G6PD expression. The authors also discovered that the PTEN protein could directly interact with G6PD and inhibit its activity through preventing the formation of a dimeric G6PD holoenzyme [52] (Figure 5a). ATM phosphorylates Hsp27 and promotes the combination of Hsp27 with G6PD, thus activating G6PD [64] (Figure 5b). These results suggest that interfering interactions between G6PD and other proteins may be an effective therapeutic approach in inhibiting cancer.

### 5.5. Other Pathways Related to G6PD in Cancer

**Figure 6:**
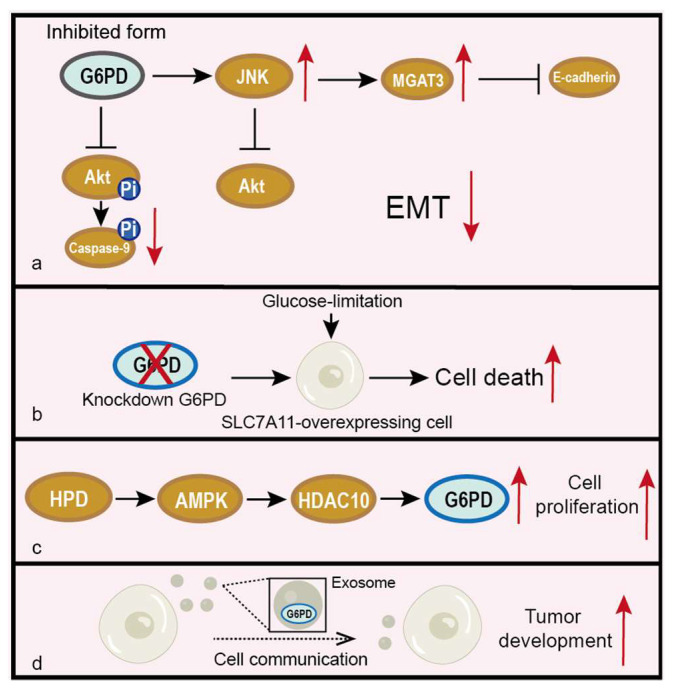
Regulation of G6PD by other pathways and their effects on tumors. Elliptical shape represents a protein. Circle shape represents post-translational modification. (**a**) Inactive G6PD will inhibit EMT through regulation of the JNK and Akt pathways. (**b**) Knockdown of G6PD promotes glucose-limitation-induced cell death in SLC7A11-overexpressing cells. (**c**) HPD promotes the expression of G6PD through the AMPK/HDAC10 pathway, thus enhancing cell proliferation. (**d**) G6PD exists in the exosome and mediates tumor development.

It has been demonstrated that G6PD regulates many signaling pathways [65,66,67]. The study of Wang et al. suggests that the inhibition of G6PD is associated with invasion, metastasis and EMT of oral squamous cell carcinoma (OSCC) through activating JNK, which enhances the E-Cadherin expression level and regulates MGAT3 expression transcriptionally to promote the bisecting GlcNAc-branched N-glycosylation of E-Cadherin [65] (Figure 6a). Chen et al. [66] revealed that the knockdown of G6PD induces intracellular apoptosis through upregulating the cleaved caspase-3, -7 and -9 levels and suppresses the phosphorylated-AKT/AKT pathway, whose activation will phosphorylate Bcl2 and inhibit apoptosis (Figure 6a). Liu et al. demonstrated that the knockdown of G6PD promotes glucose-limitation-induced cell death in solute carrier family 7 member 11 (SLC7A11)-overexpressing cells. What is more, in kidney papillary cell carcinoma (KIRP), high *SLC7A11* with high *G6PD* expression predicted a far worse clinical outcome than either parameter alone [67] (Figure 6b). Therefore, aiming to target G6PD may be a potential way to treat cancer, particularly in cases with high G6PD expression.

G6PD is also regulated by metabolic process, such as the catabolism of amino acids. Shan et al. revealed that 4-hydroxyphenylpyruvate dioxygenase (HPD) promotes a PPP flux through increasing the expression of G6PD mediated by controlling the histone acetylation levels on the *G6PD* promoter, which were mediated by the HPD-LKB1-AMPK-HDAC10 axis and increasing the acetyl-CoA levels [30] (Figure 6c).

Besides its intracellular function, G6PD may also be involved in cell-cell communications. G6PD is detected in the exosome and mediates metabolic transformation that accounts for tumor development in two late-stage ovarian cell lines, and it could act as a diagnostic, poor prognostic and therapeutic target of late-stage ovarian cancer [68] (Figure 6d).

## 6. G6PD and Chemotherapy Resistance

**Figure 7:**
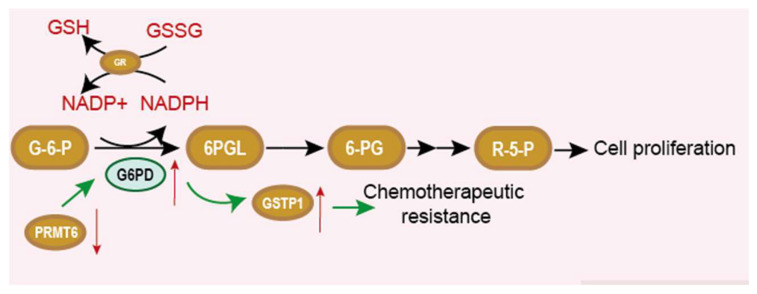
PPP pathway and the relationship between G6PD, NADPH and GSH. Elliptical shape represents a protein. Square shape represents a chemical. GR is glutathione reductase, which can transform NADPH and GSSG into NADP ^+^ and GSH. Downregulation of PRMT6 enhances G6PD expression and promotes drug resistance.

Among clinical treatments of cancer, chemotherapy is the still the standard of care for many types of cancer. At the beginning of a cure, this therapy can inhibit the growth and proliferation of cancer cells. However, the development of chemotherapy resistance frequently leads to therapy failure. Molecular mechanisms contributing to drug resistance are largely unknown, and it is urgently needed to find ways to overcome these obstacles. The inhibition of G6PD is demonstrated to be useful in reversing chemotherapy resistance. Drug resistance is related to the intracellular redox equilibrium. GSH and G6PD producing NADPH play an important role in maintaining this balance. Manuela et al. observed that the G6PD expression level and GSH level were upregulated in doxorubicin-sensitive HT29 cells. Treating cells with G6PD inhibitor dehydroepiandrosterone (DHEA) or 6-aminonicotinamide (6-AN) could decrease G6PD and GSH and reserve a multiple drug resistance [69]. This implicates that active PPP and enhanced G6PD activation are essential for MDR cells to maintain a high GSH level. What is more, another article also improved this perspective [27]. Compared to cisplatin- sensitive cells, more glutamine-derived glutamate can be used for GSH biosynthesis, and G6PD expression and activity are upregulated in cisplatin-resistant cells. Therefore, enhanced G6PD activity maybe induce the resistance of cancer cells to chemotherapeutic drugs [70]. Feng et al. provide perspective on how G6PD links to GSH and contributes to chemotherapy-resistance. Paclitaxel-resistant ovarian cancer cells showed an enhanced PPP flux in which G6PD was overexpressed. The knockdown of G6PD or using G6PD inhibitor 6-AN could improve the therapeutic efficacy of paclitaxel. Further experiments have demonstrated that the downregulation of protein arginine methyltransferase 6 (PRMT6), which methylates H3R2me2a on the promoter of G6PD cause an upregulation of G6PD. Overexpressed G6PD successively enhances the expression of glutathione S-transferase P1 (GSTP1), leading to the conjugation of GSH and paclitaxel (Figure 7). Thus, paclitaxel is exported, and its therapy efficacy is poor. The inhibition of G6PD by shRNA or 6-AN could reverse the resistance to paclitaxel [71].

## 7. Inhibitor of G6PD

**Figure 8:**
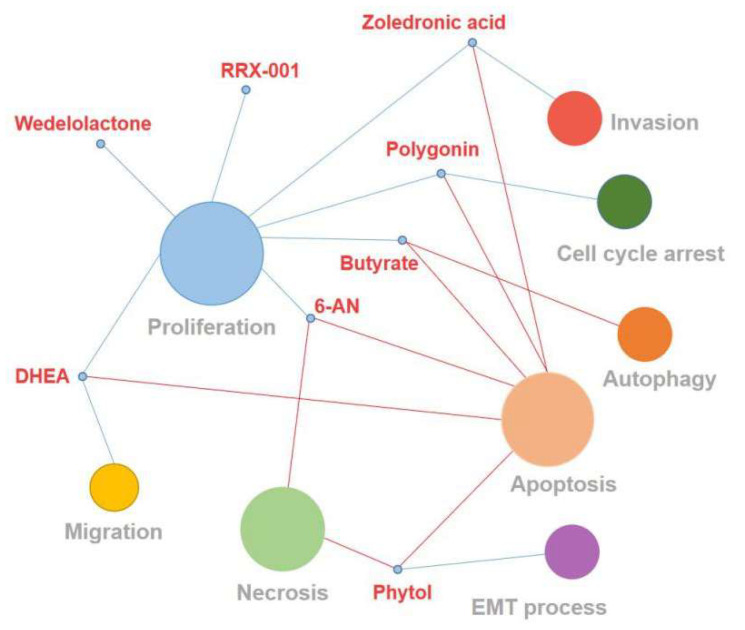
This figure shows the effect of G6PD inhibitors on cancer cellular function. The blue line represents negative regulation, and the red line represents positive regulation. The size of the circle represents the number of G6PD inhibitors with the same cellular function.

There is ample evidence that GP6D is overexpressed and promotes cancer progression in many tumors [66,72]. Thus, the inhibition of G6PD has emerged as a potential therapeutic strategy for treating cancer. The competitive G6PD inhibitor 6-AN and uncompetitive G6PD inhibitor DHEA have been widely used in various cancer studies. In addition, some natural or synthetic compounds can also inhibit G6PD (Table 2 and Figure 8).

### 7.1. 6-AN

6-AN, an analog of nicotinamide, competitively inhibits G6PD [73]. Previous studies have demonstrated that the suppression of G6PD by 6-AN can restore the sensitivity of cancer cells to chemotherapy, so it is widely used in the treatment of cancers [72]. For example, G6PD inhibition with 6-AN not only sensitizes leukemic cells to cytarabine treatment by inducing cytotoxicity against acute myeloid leukemia cells but also makes melanoma cells sensitive to metformin treatment through enhancing the cytotoxicity of metformin and resulting in increased apoptosis and necrosis [37,72]. In addition, G6PD inhibitor 6-AN can also significantly reduce the proliferation of bladder cancer cells and exert a synergistic anti-tumor effect with cisplatin [66]. However, some serious side effects such as nerve injury and vitamin B deficiency appear after 6-AN treatment [93].

### 7.2. DHEA

DHEA is an endogenous steroid produced by the adrenal glands, which acts as a metabolic precursor for the synthesis of androgen and estrogen [76]. As early as 1960, it was reported to have an uncompetitive inhibition of G6PD activity [94]. As an effective inhibitor of the G6PD targeting PPP of cancer cells, DHEA reduces the NADPH levels, NADPH-dependent oxygen-free radical production and nucleic acid synthesis to inhibit tumor development [74,95]. Fang et al. found that DHEA can inhibit the G6PD activity of cervical cancer cells, induce the apoptosis of cancer cells and lead to the decrease of cell migration and proliferation ability [75]. Yang et al. revealed that targeting G6PD by DHEA destroys the redox balance to inhibit growth in breast cancer cells [96]. Although several studies showed that DHEA has anti-cancer, anti-obesity and anti-inflammatory effects on mouse and other rodent models [97,98] DHEA is readily converted into active androgens in vivo, and clinical treatment requires a high oral dose, so its therapeutic effect is limited [94].

### 7.3. Polydatin

Polydatin (PD) is a resveratrol glycoside extracted from the root or rhizome of polygonum cuspidatum [99]. PD has many biomedical properties, such as antimicrobial, antioxidant and anti-inflammatory [99,100]. In recent years, the antitumor activity of PD has been widely investigated. Previous studies have shown that PD can suppress the growth of various cancer cells by inhibiting cell proliferation, cell cycle arrest, or inducing apoptosis [77,78]. Mele et al. reported that PD can significantly reduce tumor growth and lymph node metastases through directly inhibiting G6PD in orthotopic and metastatic models of oral cancer [94].

### 7.4. Zoledronic Acid

Zoledronic acid is a kind of bisphosphonate that is commonly used for the treatment of osteoporosis and bone metastasis in solid tumors [79,101]. Zoledronic acid has shown anticancer activity in pancreatic cancer, colon cancer and other cancers by promoting apoptosis and inhibiting cancer cell growth and invasion [79,80]. Zoledronic acid was proven to block Ras signaling and reduce the stability of TAp73, thereby inhibiting the expression of G6PD in bladder cancer cells [81].

### 7.5. Aspirin

Aspirin is a salicylic acid drug widely used for anti-inflammatory, analgesia and the prevention and treatment of cardiovascular disease [102]. In recent years, mounting studies have revealed that aspirin can inhibit many malignant tumors such as hepatobiliary and colorectal carcinoma [82]. Aspirin can acetylate a variety of proteins in cancer cells, of which G6PD is its acetylation target [36,83]. Ai et al.’s findings suggested that aspirin suppresses G6PD activity in colorectal cancer cells by acetylation, resulting in the decreased synthesis of nucleotide and NADPH [83]. Aspirin may exert anticancer effects via targeting G6PD. However, long-term use of aspirin is associated with significant risks of serious side effects, such as gastrointestinal and cerebral hemorrhage [103,104].

### 7.6. RRX-001

The dinitroazetidine derivative RRX-001 is a novel chemo-radiotherapy sensitizer in clinical [36,105]. RRX-001 can increase the sensitivity of the tumor to radiation and reverse the drug resistance properties to improve the therapeutic efficiency of chemo-radiotherapy [84]. RRX-001 has a significantly G6PD activity inhibition effect on cancer cell lines, including HepG2, CACO-2 and HT-29 [106]. Cell proliferation is further inhibited by increasing the reactive oxygen/nitrogen species and decreasing ribose 5-phosphate synthesis and glutathione [106,107]. Therefore, RRX-001 as an inhibitor of G6PD for cancer treatment is particularly promising.

### 7.7. Phytol

A number of compounds have been used to inhibit the G6PD activity in vitro and in vivo. Parth et al. found that phytol has a good binding affinity with G6PD and could also inhibit the activity of G6PD during in silico molecular docking studies [108]. Natural product phytol is an acyclic monounsaturated diterpene alcohol that widely exists in algae, plants and bacteria [85,109]. Previous research has confirmed that phytol represents a wide range of biological activities, such as an antioxidant effect, cytotoxicity and anti-inflammatory and antimicrobial activities. It is worthwhile mentioning that phytol can induce the apoptosis and necrosis of various cancer cells and inhibit the EMT process in HepG2 cells but have little toxicity to normal cells, suggesting that phytol exhibits considerable anticancer potential [85,86]

### 7.8. Wedelolactone

Wedelolactone, a small molecular compound isolated from Ecliptaalba and Wedelia calendulacea has attracted much attention because of its extensive pharmacological activities, including hepatoprotective, anti-oxidation, anti-inflammatory, immunomodulatory and anti-fibrotic effects, etc. [87,110,111,112] Recent researches has shown that Wedelolactone exhibited a potent anticancer activity against different solid tumors, such as breast, colon and prostate cancers, through inhibiting multiple kinases, the androgen receptor, 5-lipoxygenase and the c-Myc protein [87]. Lately, Luo et al. identified Wedelolactone as a G6PD inhibitor by the high-throughput screening assay. The study demonstrated that it could not only inhibit G6PD significantly in a non-competitive and reversible way but also suppress the proliferation of ovarian cancer cells, which suggests that Wedelolactone affects the proliferation of ovarian cancer cells partly through blocking the enzyme activity of G6PD [88].

### 7.9. Butyrate

Butyrate is one of the short-chain fatty acids produced by the gut microbiota along with acetic acid and propionic acid. Studies have shown that butyrate exerts several effects in normal colon cells, including supplying energy, maintaining the gut barrier and reducing inflammation [113,114,115], while it exhibits anti-tumorigenic activities through the induction of apoptosis, inhibition of proliferation and promotion of autophagy in various cancer types [89,90,91]. A recent study showed that butyrate can down-regulate the G6PD mRNA and protein levels, reduce the DNA synthesis activity and further enhance the apoptosis efficacy of 5-FU on colon cancer cells. It is reported that butyrate could also inhibit histone deacetylases (HDACs) to depress a number of oncogenic signaling pathways [92]. As a dual inhibitor of G6PD and HDAC, butyrate has great potential to become an anticancer drug in the future.

## 8. Discussion

Metabolism is necessary for all organisms and cells depend on which provide nutrients, protecting cells from stressed environments and meeting the demands of life. However, increasing evidence has indicated that the metabolic pathways of cancer cells are changed compared to normal cells. These alterations enable cancer cells to acquire more energy, maintain excessive life demands or even transform into malignant cells and improve their ability to translocate and escape from immunological surveillance. Therefore, identifying additional metabolic changes of cancer cells and uncovering efficient methods to modulate these changes may lead to novel cancer treatment. PPP as a glucose metabolism pathway is essential in synthesizing nucleotides and providing NADPH. Targeting the PPP in tumors is an effective approach in treating cancer and improving therapeutic efficacy. Dysfunctions of enzymes in this pathway such as 6PGD contribute to the development of cancer [116,117,118]. Expression changes of G6PD also promote cancer development. The formation of malignant cancer needs many procedures. The beginning of cancer cells stems from uncontrolled growth and continuous replication. Some of cancer cells then evolve the abilities of metastasis and invasion. All of these steps are complicated and involve many pathways. To be noticed, G6PD promotes many of these steps not only as a regulator but also as an actor. In most cancers, G6PD expression is upregulated. The overexpression of G6PD helps cancer cells in their growth, proliferation, EMT, metastasis and invasion. From the normal role of G6PD, we can easily find that the overexpression of G6PD will produce more NADPH and nucleotides. As we all know, NADPH protects cells from life stress, which cancer cells confront a lot. Besides, producing nucleotides can meet the demand of repaid growth and fast proliferation. All of these provide cancer cells with favorable condition to live. Therefore, it is not a surprise that G6PD is involved in the pathways that regulate the cell cycle, expression of cadherin, DNA synthesis and DNA repair. According to several previous studies, the regulations on G6PD depend on multiple aspects from the transcriptional level to the post-translational modification level. Interactions with other proteins could also impact on the functions of G6PD. What is more, the mechanisms regulating G6PD and the pathways regulated by G6PD impact each other and form a circulation. This implied that networks regulating cancer cells are complicated, and G6PD is a promising target. The reverse abnormal expression of G6PD has shown positive effects in inhibiting cancer. However, the roles of G6PD in cancer development mainly depend on its metabolic function in producing NADPH to reduce ROS. Whether G6PD contributes to the development of cancer independent of its metabolic and enzyme function needs to be further discussed. The development of drug resistance is the main cause contributing to the failure of chemotherapy, which is tightly related to the prognosis and survival of patients. Inhibiting G6PD could reverse the chemo-resistance. PPP produces NADPH so that cancer cells with hyper PPP activity have a different intracellular redox system, and this makes drug-resistant cells less susceptible to oxidative stress induced by chemotherapeutic drugs. Reversed chemo-resistance was achieved in the experiment performed by Hong et al., Feng et al. and Yang et al. Based on the essential functions in developing cancer, finding a G6PD inhibitor may be an effective way of treating cancer. Several inhibitors have been found and been proven to suppress the proliferation of cancer cells. However, problems still exist. According to scientific reports, DHEA is an endogenous steroid hormone, and it is a strong noncompetitive inhibitor that can suppress G6PD. Besides, it is the only G6PD inhibitor that was used in vivo. Though DHEA could induce the apoptosis of cancer cells through G6PD, clinical trials of DHEA have been hampered by the high oral dose required and the hindering in the conversion of DHEA to active androgen. Therefore, its effectiveness as a G6PD inhibitor remains controversial. Other chemicals such as polygonin, zoledronic acid, RRX-001, Phytol, 6-AN, ect. inhibit G6PD activity or reduce the expression level of G6PD as well. However, their effectiveness in vivo and whether they can be further used in clinical are still unknown. What is more, whether these inhibitors are also efficient in tumors with low G6PD expression, and whether these inhibitors can reverse the chemoresistance of the current clinical drugs in treating cancer are questions that need to be further explored. Developing more potential drugs aimed at G6PD is a promising but still long and rough way in cancer treatment.

In conclusion, this review described the history and structure of G6PD, the effects of high G6PD expression (or high activity) on cancer, the regulation of G6PD expression from different aspects, and the role of G6PD in chemoresistance. However, the regulated mechanisms linked to G6PD are complicated and not fully understood; thus, which mechanism is most important in cancer development is still unknown. Further studies are necessary to reveal more possibilities of G6PD in cancer because of its important role in both providing biosynthesis material and NADPH for redox equilibrium.

## Figures and Tables

**Table 1:** G6PD expression in different cancers and their effects on cancer.

Cancer Type	G6PD Expression	Effects of High/Low Expression
Lung adenocarcinoma [34]	High	Advanced stage, Metastasis, Invasion
Glioma [35]	High	Poor survival
Colorectal cancer [36]	High	Poor prognosis, Poor outcome of chemotherapy
Acute myeloid leukemia [37]	Low	Reduced chemotherapy-resistance
Clear cell renal cell carcinoma [29]	High	Invasion and ECM
Breast carcinoma [38]	High	Chemotherapy-resistance
Esophageal squamous cell carcinoma [39]	High	Metastasis, Poor survival
Hepatocellularcarcinoma [33,40]	High	Growth, Invasion, ECM, Migration
Gastric cancer [27]	High	Invasion, ECM, Migration, Poor survival
Melanoma [41]	Low	Decreased proliferation, Enhanced apoptosis
Pituitary cancer [42]	Low	Decreased glycolysis

**Table 2:** G6PD inhibitors in different cancer(cell) types.

Compounds	Cancer (Cell) Type	Reference
6-aminonicotinamide (6-AN)	Acute myeloid leukemia cells, melanoma cells, bladder cancer cells	[66,72,73]
Dehydroepiandrosterone (DHEA)	Cervical cancer cells, breast cancer cells	[74,75]
Polygonin	Lung cancer cells (A549 and NCI-H1975), nasopharyngeal carcinoma cells, epidermal carcinoma cells (A-431), breast cancer cells (MCF-7), ovarian cancer cells (OVCAR-8), cervical carcinoma cells (HeLa), osteosarcoma cells, orthotopic and metastatic model of oral cancer (mice)	[76,77,78]
Zoledronic acid	Lung cancer (patients), orthotopic implantation model of pancreatic cancer (mice), mammary cancer (mice), pancreatic cancer cells, bladder cancer cells	[79,80,81]
Aspirin	Hepatobiliary and colorectal carcinoma (patients), colorectal cancer cells	[36,82,83]
RRX-001	Colorectal cancer cells (CACO-2, HT-29), hepatoma cells (HepG2)	[84]
Phytol	Hepatoma cells (HepG2), breast cancer cells (MCF-7, MDA-MB-231), prostate cancer cells (PC-3), colorectal cancer cells (HT-29), lung cancer cells (A-549), melanoma cells (Hs294T)	[85,86]
Wedelolactone	Ovarian cancer cells (Ovcar3), breast cancer cells (MDA-MB-231), colon cancer cells (WiDr), prostate cancer cells	[87,88]
Butyrate	Colon cancer cells (HCT116, LoVo, SW480, HT29)	[89,90,91,92]

## Data Availability

Not applicable.
